# Development of Triticale × Wheat Prebreeding Germplasm With Loci for Slow-Rusting Resistance

**DOI:** 10.3389/fpls.2020.00447

**Published:** 2020-05-07

**Authors:** Roksana Skowrońska, Monika Mariańska, Waldemar Ulaszewski, Agnieszka Tomkowiak, Jerzy Nawracała, Michał T. Kwiatek

**Affiliations:** ^1^Department of Genetics and Plant Breeding, Poznań University of Life Sciences, Poznań, Poland; ^2^Institute of Plant Genetics of the Polish Academy of Sciences, Poznań, Poland

**Keywords:** genomic *in situ* hybridization, molecular markers, resistance genes, slow rust, triticale, wheat

## Abstract

There is a growing interest in breeding and production of hexaploid triticale (× *Triticosecale* Wittmack ex A. Camus) in European Union and in the world. It is reported that triticale can be an alternative to wheat (*Triticum aestivum* L.) for livestock feed production and has a potential to become preferred industrial energy crop. Fungal diseases, mainly leaf and stripe rusts, are the limiting factors of triticale growth and yield. Geneticists and breeders are now focusing on accumulation of the major genes for durability of rust resistance. Slow-rusting genes *Lr34/Yr18* and *Lr46/Yr19* are being exploited in many wheat breeding programs. This type of horizontal resistance is reported to be effective over space and time. Classical breeding techniques supported by marker-assisted selection (MAS) are the main tools in breeding programs. The aim of this study was to assess the possibility of transfer of slow-rusting genes from resistant genotypes of wheat into hexaploid triticale through cross-hybridizations. A total of 5,094 manual pollinations were conducted between two triticale cultivars Fredro and Twingo and 33 accessions of common wheat, which were reported as sources of slow-rusting resistance genes. The investigation of the slow-rusting gene transmission was performed using both molecular markers analyses and genomic *in situ* hybridization (GISH). In total, 34 F_1_ hybrid plants were obtained, and 29 of them carried both slow-rusting loci. Therefore, these hybrids may be used for triticale prebreeding program.

## Introduction

Triticale (× *Triticosecale* Wittmack ex A. Camus) is an artificial manmade grain, created through hybridization of wheat derivatives (*Triticum* sp.) with rye (*Secale cereale* L.). Initially, triticale was expected to combine the high robustness of rye with the great yield potential (Kwiatek and Nawracała, 2018). At present, this crop is widely used as an alternative for other cereals, mostly for wheat, as a valuable source of livestock feed (Ayalew et al., 2018). In recent years, the interest in triticale production has increased because of its potential to become an industrial energy crop (Sanaei and Stuart, 2018). The economic importance of triticale is reflected by a significant acreage in Europe, which accounts for 90% of the world production (FAO, 2018). Triticale, as an artificial crop, is characterized by low genetic variation. Moreover, the increasing harvesting area of this crop is associated with the rapid development of fungal pathogens, which are continuously adapting to triticale. Hence, the need to develop more basic and applied research on this crop connected with resistance breeding.

Leaf rust caused by *Puccinia triticina* Eriks. and stripe rust caused by *Puccinia striiformis* Westend f. sp. *tritici* are the most important foliar diseases of triticale, as well as other major cereal species. It is reported that rye genome in triticale improves the resistance for leaf rust (Mikhailova et al., 2009). Genetic resistance is the most economical and preferable method of reducing yield losses due to leaf rust (Kolmer, 1996). More than 70 leaf rust resistance genes (*Lr*) have been mapped to specific chromosomes and described in the Catalog of Gene Symbols for Wheat (McIntosh, 1995; McIntosh et al., 2016). Many of the leaf and stripe rust resistance genes are race specific and have been overcome by the new races of *Puccinia* spp. (Periyannan et al., 2017). However, in the wheat genepool, three genes were identified that confer durable adult plant resistance against multiple fungal diseases (Ellis et al., 2014). These genes were named *Lr34* (= *Yr18/Sr57/Pm38*; chromosome 7D), *Lr46* (= *Yr29/Sr58/Pm39*; 1B), and *Lr67* (= *Yr46/Sr55/Pm46*; 4D) (Dyck, 1977; Dyck and Samborski, 1979; Singh et al., 1998). Their expression results in partial resistance against all races of the fungal wheat pathogens causing leaf rust, stripe rust, and powdery mildew (*Blumeria graminis* f. sp. *tritici*).

New plant-breeding techniques (NPBTs) developed over the past two decades have provided comprehensive opportunities for efficient trait development in crops (Eriksson et al., 2018). For example, transgenic barley (*Hordeum vulgare* L.) lines expressing slow-rusting allele *Ta-Lr34res* of wheat showed enhanced resistance against leaf rust and powdery mildew of barley (Boni et al., 2018). Unfortunately, diverging opinions and politically motivated arguments are hampering the political progress to decide on the regulation of NPBTs in the European Union. Hence, classical breeding techniques, such as wide cross-hybridization supported by marker-assisted selection (MAS), are still the main tool for breeding programs.

The aim of this work was to assess the possibility of transfer of two main slow-rusting genes *Lr34* and *Lr46* from wheat-resistant genotypes derived from the United States Department of Agriculture (USDA)/Agricultural Research Service (ARS) Small Grains Laboratory, Aberdeen (ID, United States) gene bank into two elite cultivars of hexaploid triticale through cross-hybridizations. The evaluation of the chromosome transmission was performed using both molecular marker analyses and genomic *in situ* hybridization (GISH). This initial study is to obtain the starting pool triticale–wheat hybrids, carrying slow-rusting genes, which will be applied in the triticale breeding programs.

## Materials and Methods

### Plant Material

Seeds of 33 accessions of wheat, which were reported as sources of slow-rusting genes (Table 1), and two cultivars of winter triticale were germinated on Petri dishes. The plantlets were transferred to soil and cultivated for 6–8 weeks under short-day conditions (8 h light/16 h dark, 20/18°C). Finally, the winter genotypes were transferred for 6 weeks to vernalizing conditions (10 h light/14 h dark, 4°C) and then returned to long-day conditions (13 h light/11 h dark, 20/16°C).

**TABLE 1:** Pollen viability of wheat accessions and seed production ability of triticale after pollination with pollen grains from wheat components.

No.	Pollinators (*Triticum aestivum* L.)								Maternal components (× *Triticosecale* Wittmack)					
		
	Cultivar/genotype	Plant ID	Growth habit	Source	*csLV34* linked to *Lr34*	*Xgwm44* linked to *Lr46*	*csLV46G22* linked to *Lr46*	Pollen viability (%)	Triticale cv. “Fredro” (Danko			Triticale cv. “Twingo” (Danko		
										
									Breeding Company, Poland)			Breeding Company, Poland)		
									Number of pollinated flowers	Number of hybrid seeds	Crossing efficiency (%)	Number of pollinated flowers	Number of hybrid seeds	Crossing efficiency (%)
1.	Frontana	Cltr 12470	Spring	USDA	+	+	+	90.29	62	0	0.00	124	0	0.00
2.	Chris	Cltr 13751	Spring	USDA	+	+	−	99.36	68	0	0.00	350	5	2.86
3.	H_N_ROD6_13751	PI 191772	Spring	USDA	−	−	−	89.33	*not crossed*			*not crossed*		
4.	Frontana 3671	PI 193932	Spring	USDA	+	+	+	92.23	56	0	0.00	56	12	21.43
5.	Frontana LF 320	PI193933	Spring	USDA	+	+	+	88.29	58	0	0.00	116	0	0.00
6.	Frontana LF 321	PI193934	Spring	USDA		+	+	88.74	52	2	3.85	100	2	2.00
7.	Fronthatch-1	PI 290745	Spring	USDA	+	+	+	89.36	60	0	0.00	64	0	0.00
8.	Fronthatch-2	PI 297014	Spring	USDA	+	+	+	89.09	56	0	0.00	66	0	0.00
9.	Fronthatch-3	PI 299419	Spring	USDA	+	+	−	88.92	62	0	0.00	58	0	0.00
10.	Toropi	PI 344200	Spring	USDA	−	−	+	90.34	*not crossed*			*not crossed*		
11.	Fortaleza	PI 351779	Spring	USDA	+	+	−	89.74	64	0	0.00	56	0	0.00
12.	Sparrow	PI 519725	Spring	USDA	+	+	+	91.24	62	0	0.00	58	1	1.72
13.	Pavon F76	PI 519847	Spring	USDA	−	+	+	89.60	88	0	0.00	100	0	0.00
14.	Pavon 76	PI 520003	Spring	USDA	−	+	−	89.36	64	0	0.00	64	0	0.00
15.	Pavon-1	PI 520054	Spring	USDA	+	+	+	89.81	68	0	0.00	48	0	0.00
16.	Pavon-2	PI 520172	Spring	USDA	−	+	+	88.24	62	0	0.00	64	0	0.00
17.	Myna	PI 520340	Spring	USDA	+	+	−	90.46	58	0	0.00	68	0	0.00
18.	Junco	PI 519947	Spring	USDA	−	−	−	88.10	*not crossed*			*not crossed*		
19.	Tanager	PI 519878	Spring	USDA	−	+	+	90.13	62	0	0.00	56	0	0.00
20.	Parula	PI 520340	Spring	USDA	+	−	−	89.36	68	0	0.00	170	0	0.00
21.	Rayon 89	PI 591786	Spring	USDA	−	−	−	95.81	*not crossed*			*not crossed*		
22.	Cumpas 88	PI 591786	Spring	USDA	+	−	+	91.20	58	0	0.00	62	0	0.00
23.	Mochis 88	PI 591791	Spring	USDA	+	−	−	94.83	54	0	0.00	160	9	13.13
24.	P8901-AP	PI 613175	Spring	USDA	+	−	−	89.19	56	0	0.00	64	0	0.00
25.	P8901-AQ	PI 613176	Spring	USDA	+	−	−	91.08	64	0	0.00	64	0	0.00
26.	Tlaxcala F2000	PI 619634	Spring	USDA	+	+	+	89.34	54	0	0.00	68	0	0.00
27.	Lr34	GSTR 433	Spring	USDA	+	−	−	89.41	62	0	0.00	58	0	0.00
28.	IWA8608696	PI 624623	Spring	USDA	+	+	−	88.34	58	0	0.00	56	0	0.00
29.	Anza	PI 638742	Spring	USDA	+	+	−	90.91	56	0	0.00	62	0	0.00
30.	UC1110	PI 671999	Spring	USDA	+	+	−	89.63	64	0	0.00	128	0	0.00
31.	Kern	PI 672001	Spring	USDA	+	+	−	88.59	68	0	0.00	164	0	0.61
32.	TX89D6435	PI 584759	Winter	USDA	+	+	−	93.70	62	0	0.00	658	3	0.46
33.	Purdue	Cltr 13227	Winter	USDA	+	+	+	91.13	68	0	0.00	138	0	0.00
	Mean		90.46	61.86	0.07	0.001	113.79	1.10	0.01					
	Total		n/a	1,794	2	n/a	3,300	32	n/a					

### Pollen Viability Evaluation and Cross-Hybridizations

F_1_ hybrid plants were obtained through cross-hybridization between triticale (female parent) and wheat (pollinator) performed in the glasshouse chambers of the Department of Genetics and Plant Breeding at the Poznań University of Life Sciences (PULS), Poland. In this purpose, 10 seeds from each accession were sown in plastic pots in three replications. Evaluation of pollen vitality was made on the wheat genotypes. Pollen grains were stained with 2% aceto-carmine in glycerine (vol. 1:1) for the presence of cytoplasm. The evaluation of pollen vitality was made using a Delta Genetic Pro microscope (Delta Optical, Poland).

Florets of maternal components (triticale plants) were emasculated to avoid self-fertilization in order to cross with the pollen of wheat. The emasculated florets were counted and pollinated with freshly collected pollen of wheat within a period of 3 months (April–June 2019). The percentage ratio of the total amount of seeds from each plant with the total amount of pollinated flowers of each plant was calculated [crossing efficiency (CE)].

### Identification of Molecular Markers Linked to *Lr34* and *Lr46* Genes

The following molecular markers *csLV34* (Lagudah et al., 2006) linked to *Lr34* and *Xgwm44* (William et al., 2003) and *csLV46G22* (Lagudah pers. comm.) linked to *Lr46* were used to confirm the presence of alleles connected with slow-rusting resistance in wheat genotypes and F_1_ hybrids. Genomic DNA was extracted from seedling leaves using the Plant and Funghi DNA Purification Kit (EURx, Poland). The PCR reaction volume was 20 μl, consisting of 100 nM each of the two primers, 2 × TaqNova-DNA hot-start polymerase buffer (Blirt, Poland), and 50 ng of genomic DNA as template. A typical PCR procedure was as follows: 5 min at 95°C, then 35 cycles of 30 s at 94°C, 30 s at 50–60°C (Skowrońska et al., 2019), 1 min at 72°C, and 5 min at 72°C. PCR products were run on 2% agarose gel (Lab Empire, Poland) with 1% Tris–borate–ethylenediaminetetraacetic acid (TBE) buffer. DNA was visualized via Midori Green Direct (Nippon Genetics Europe, Germany) that was added to the samples.

### Genomic *in situ* Hybridization

Chromosome sets of F_1_ hybrids were analyzed using **GISH**. This approach was to calculate the cross-hybridization efficiency (CE). It was performed on mitotic chromosomes of root meristem cells collected from hybrid plants. Mitotic metaphase accumulation and fixation procedures were carried out according to Kwiatek et al. (2016). Total genomic DNA was isolated using the DNeasy Plant Maxi Kit 24 (Qiagen, Germany). DNA of *Aegilops tauschii* Coss. (2*n* = 2*x* = 14 chromosomes; DD; PI 603226; US National Plant Germplasm System), a progenitor of the D-genome of wheat, was labeled by nick translation with Atto-488 dye (Atto-488NT kit; Jena Bioscience, Germany) for the investigation of D-genome chromosomes. Total genomic DNA of rye (Imperial; PI 323382; US National Plant Germplasm System) was labeled in the same way by Atto-550 dye. Blocking DNA from *T. durum* Desf. (2*n* = 4*x* = 28 chromosomes; AABB; Ceres; HR Smolice; Poland) was sheared by boiling for 30–45 min and used at a ratio of 1:50 (probe/block). **GISH** was performed according to Kwiatek et al. (2016). Slides were analyzed with the use of an Axio Observer 7 (Carl Zeiss, Oberkochen, Germany) fluorescence microscope. Image processing was done using ZEN Pro software (Carl Zeiss, Oberkochen, Germany). Each plant was evaluated by an analysis of chromosome sets of 10 cells.

## Results

### Identification of Molecular Markers Linked to Loci of Slow-Rusting Genes in Wheat Accessions

**FIGURE 1:**
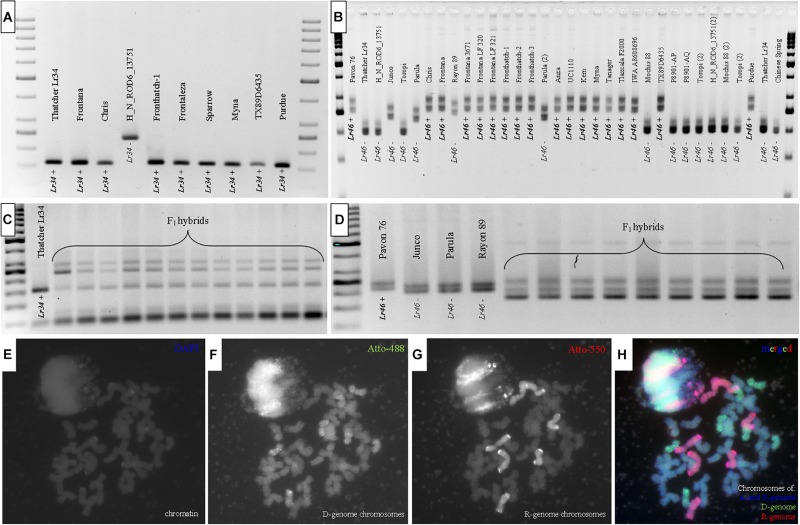
Amplification products of PCR reactions of **(A)** wheat accessions (pollen donors) and **(C)** F_1_ hybrids with *csLV34* marker linked to *Lr34* (= *Yr18/Sr57/Pm38*) locus and **(B)** wheat accessions and **(D)** F_1_ hybrids with *Xgwm44* marker linked to *Lr46* (= *Yr29/Sr58/Pm39*). *Lr34* or *Lr46+* indicate resistant alleles; *Lr34-* or *Lr46–* indicate susceptible alleles. Genomic *in situ* hybridization preformed on mitotic chromosomes of plants derived through cross-hybridization between triticale and wheat: **(E)** chromatin (DAPI), **(F)** D-genome chromosmes (Atto-488, green), **(G)** R-genome chromosomes (Atto-550, red), **(H)** merged.

The molecular identification of *Lr34* and *Lr46* alleles was performed in all wheat and triticale cultivars and genotypes using *csLV34* and *Xgwm44* markers, respectively. The Thatcher near-isogenic line Lr34 (*Lr34*; GSTR 433; USDA) and Pavon 76 (*Lr46*; PI 520003; USDA) lines were used as a positive control (R; Figures 1A,B). The PCR reactions using *csLV34* resulted in the amplification products of 160 bp in size and were found in DNA extracts of Lr34-positive control (GSTR 433) and 24 wheat cultivars and genotypes (Table 1). A PCR product of 240 bp in size was identified for eight wheat genotypes, indicating susceptible (S) allele (Figure 1A and Table 1). PCR reaction with *Xgwm44* marker yielded two amplicons: 260 and 280 bp for a product for Pavon76 and 17 wheat accessions (Figure 1B). Eleven wheat genotypes were characterized by 200 and 230 bp bands. Two wheat accessions showed 240- and 270-bp bands. “Toropi” wheat showed 235- and 265-bp band pattern (S-allele). The second *Lr46* marker, *csLV46G22*, gave different results, compared to *Xgwm44*. R-allele was identified in 16 wheat accessions. The results of *Xgwm44* and *csLV46G22* markers were similar for 21 of 33 wheat accessions. Ten wheat accessions carried all three resistant allele markers for *Lr34* (*csLV34*) and *Lr46* (both *Xgwm44* and *csLV46G22*) (Table 1).

### Pollen Viability of Wheat Accessions With Slow-Rusting Genes

The viability of pollen grains of wheat accessions was high and ranged between 88.29 and 99.36% (Table 1). The highest pollen viability was observed for “Chris” wheat cultivar. The lowest viability of pollen grains was evaluated in “Junco” cultivar. The mean pollen viability evaluated for all 33 wheat accession used for this experiment amounts to 90.45%. Following wheat accessions: Frontana, Frontana 3671, Toropi, Sparrow, Myna, Tanager, Rayon 89, Mochis 88, P8901-AQ, Anza, TX89D6435, and Purdue showed high viability of pollen grains (in excess of 90%).

### Evaluation of Triticale × Wheat Cross-Hybridizations

Cross-hybridization of winter triticale with wheat slow-rusting pollinators was performed in order to evaluate the CE through triticale × wheat crossing combinations (Table 1). Total amount of 5,094 flowers of triticale were pollinated with wheat pollen grains. The crossing evaluation showed, that in general, triticale cv. Twingo produced more hybrid seeds (32 seeds) than cv. Fredro (two seeds). Overall, hybrid seeds were provided by only seven crossing combinations. The total CE for both triticale cultivars was surprisingly low and amounts to 0.1% for Twingo × wheat combinations and 0.001% for Fredro × wheat combinations. Twingo × Frontana 3671 cross-hybridization was the most effective and yielded 12 seeds (CE, 21.43%).

### Identification of Molecular Markers Linked to Loci of Slow-Rusting Genes in Triticale × Wheat F_1_ Hybrids

Similar set of three molecular markers, *csLV34*, *Xgwm44*, and *csLV46G22* was used to screen the presence of *Lr34* and *Lr46* loci in F_1_ hybrids (H) (Figure 1C and Table 2). The PCR reactions for *csLV34* marker yielded three types of amplicons: 160 bp from resistant wheat (chromosome 7D) and 250 and 300 bp from R-genome of triticale (Figure 1C). The evaluation of electropherograms generated after separation of PCR products, which were amplified using *Xgwm44* marker, was more difficult. Three products were amplified using DNA samples from F_1_ hybrids (H): 150, 200, and 240 bp, which were characteristics for both 1B chromosome that originated from resistant wheat accessions and 1B chromosome from triticale (Figure 1D). The resistant allele of *csLV46G22* marker was identified in plants of four crossing combinations. Moreover, those combinations (Fredro × Frontana LF 321, Twingo × Frontana LF 321, Twingo × Frontana 3671, and Twingo × Sparrow) carried all three resistant allele markers linked to *Lr34* and *Lr46* slow-rusting genes (Table 2).

**TABLE 2:** Chromosome sets of triticale × wheat hybrids and the identification of marker loci linked to slow-rusting genes.

No.	Crossing combination	F_1_ plant ID	Number of chromosomes	Number of A- and B-genome chromosomes	Number of R-genome chromosomes	Number of D-genome chromosomes	*csLV34* linked to *Lr34*	*Xgwm44* linked to *Lr46*	*csLV46G22* linked to *Lr46*
1.	Fredro × Frontana LF 321	1	42	28	7	7	+	+	+
2.		2	42	28	7	7	+	+	+
3.	Twingo × Chris	1	42	28	7	7	+	+	−
4.		2	42	28	7	7	+	+	−
5.		3	42	28	7	7	+	+	−
6.		4	42	28	7	7	+	+	−
7.		5	42	28	7	7	+	+	−
8.	Twingo × Frontana 3671	1	42	28	7	7	+	+	+
9.		2	42	28	7	7	+	+	+
10.		3	42	28	7	7	+	+	+
11.		4	42	28	7	7	+	+	+
12.		5	42	28	7	7	+	+	+
13.		6	42	28	7	7	+	+	+
14.		7	42	28	7	7	+	+	+
15.		8	42	28	7	7	+	+	+
16.		9	42	28	7	7	+	+	+
17.		10	42	28	7	7	+	+	+
18.		11	42	28	7	7	+	+	+
19.		12	42	28	7	7	+	+	+
20.	Twingo × Frontana LF 321	1	42	28	7	7	+	+	+
21.		2	42	28	7	7	+	+	+
22.	Twingo × Sparrow	1	42	28	7	7	+	+	+
23.	Twingo × Mochis 88	1	42	28	7	7	+	−	−
24.		2	42	28	7	7	+	−	−
25.		3	42	28	7	7	+	−	−
26.		4	42	28	7	7	+	−	−
27.		5	42	28	7	7	+	−	−
28.		6	42	28	7	7	+	−	−
29.		7	42	28	7	7	+	−	−
30.		8	42	28	7	7	+	−	−
31.		9	42	28	7	7	+	−	−
32.	Twingo × TX89D6435	1	42	28	7	7	+	+	−
33.		2	42	28	7	7	+	+	−
34.		3	42	28	7	7	+	+	−

### Chromosome Constitution of F_1_ Hybrids Revealed by Genomic *in situ* Hybridization

Evaluation of chromosome constitution was made for all 34 F_1_ seeds in order to confirm their hybrid origin (Figures 1E–H and Table 2). The karyotype for triticale × wheat F_1_ hybrid contained 14 chromosomes (seven pairs) of A-genome, the same amount of B-genome chromosomes, seven chromosomes (monosomic) belonging to D-genome (Figure 1F), which were derived from wheat parent, and seven monosomic chromosomes of R-genome derived from triticale parent (Figure 1G).

## Discussion

Development of transgenic triticale as a source of bioindustrial products is currently one of the main challenges for breeders. For now, pollen-mediated gene flow from related species into conventional triticale varieties is the only pathway for transgene movement in large-scale breeding programs leading in the European Union. Currently, most breeders lean toward the use of slow-rusting genes for durable and race non-specific resistance of cereals. The main aim of this study was to obtain the starting pool triticale–wheat hybrids, which will be further used for backcrossing program, evaluation of slow-rusting gene expression, and inoculation tests.

The *Lr34* gene has been widely used in breeding of wheat cultivars worldwide, and its resistance has remained effective over many years despite large-scale agricultural use. The gene was identified in several distinct groups of genetic material, e.g., CIMMYT lines, Chinese landraces, and European winter wheat germplasm, for example Frontana, which was developed in Brazil or the Italian wheat Mentana (Kolmer et al., 2013). Lagudah et al. (2009) developed a sequence-tagged site (STS) marker, *csLV34*, that maps 0.4 cM from *Lr34 locus* and was validated in many lines and cultivars from different breeding programs worldwide. The *Lr34* allele yielded a 150-bp product, and a 229-bp band was amplified in non-Lr34 germplasm (Lagudah et al., 2009). In this study, the product for resistant genotypes of wheat was the same. A 230-bp band was amplified for wheat accessions with the lack of *Lr34* allele (Rod, Toropi, Junco, etc.). However, different bands were amplified for susceptible triticale cultivars and F_1_ hybrids (250 and 350 bp). Hence, it could be assumed that the *csLV34* ortholog is placed on R-genome chromosomes (probably on 7R chromosome) but yields different amplicons, which are useful for identification of *Lr34* locus in triticale × wheat hybrids.

The second major slow-rusting gene, *Lr46*, was first described in 1998 by Singh et al. (1998) in Mexican cultivar Pavon 76 and located on chromosome 1B (William et al., 2003). Suenaga et al. (2003) determined that the microsatellite locus *Xwmc44* is located 5.6 cM proximal to the putative quantitative trait locus (QTL) for *Lr46*. However, it is reported that the *Xwmc44* resistance allele in some cases is not diagnostic of *Lr46*, since numerous varieties without *Lr46* have *Xwmc44* products of similar sizes (Dubcovsky and Soria, 2017). In this study, *Xwmc44* marker yielded four different amplification products for F_1_ hybrids. The size differences between amplicons were difficult to evaluate by standard electrophoresis using 2% agarose gel. In comparison, we have used *csLV46G22* marker (primer sequences and protocols were kindly provided by Prof. E. Lagudah, CSIRO, Australia), which is highly reliable and close to 100% diagnostic marker for the *Lr46* gene (Lagudah, personal communication). This cleaved amplified polymorphic sequence (CAPS) marker is codominant and appeared to be useful for genotyping on the F_1_ hybrids. Moreover, we showed the poor diagnostic ability of the *Xwmc44* marker used to trace the *Lr46* gene. Moreover, we identified mismatch results between *Xgwm44* and *csLV46G22* in 12 of 33 wheat accessions.

The GISH experiment confirmed the presence of seven monosomic chromosomes of D-genome derived from wheat accessions in most of the seeds obtained from cross-hybridizations of triticale and wheat in this study. This method has direct applications on the fundamental research, as well as in detecting the amount of introgressed chromatin during the production of prebreeding germplasm (Schwarzacher et al., 1992; Kwiatek et al., 2019). A number of reports showed the ability of cytogenetic methods to determine the introgression of D-genome chromosomes of wheat or *A. tauschii* Coss. into triticale genetic background (Lukaszewski et al., 1987; Salmanowicz et al., 2013; Kwiatek et al., 2015). The *Lr34* locus is located on 7D chromosome of slow-rusting wheat accessions; hence, GISH was an additional method to prove the hybrid origin of F_1_ plants and to confirm the transfer of D-genome chromosomes with *Lr34* locus into hybrid plants, *per se*. Unfortunately, *Lr46* locus identification cannot be supported by GISH because this method is not a applicable to discriminate B-genome chromosomes of F_1_ hybrids, which were originated from both wheat and triticale parental forms.

A combination of MAS and GISH enabled to select triticale × wheat hybrid plants with loci slow-rusting genes. Twingo × Frontana 3671 hybrids seems to be the most promising prebreeding forms, considering the accumulation of both *Lr34-* and *Lr46*-resistant alleles. Those combinations will be used for seed propagation, further backcrossing, and resistance evaluation. The crucial approach for this research is to improve the crossability of triticale cultivars with chosen wheat pollinators. What is more, field and greenhouse resistance tests will be essential to confirm whether the transferred slow-rusting genes will provide resistance in the triticale background.

## Data Availability Statement

The raw data supporting the conclusions of this article will be made available by the authors, without undue reservation, to any qualified researcher.

## Author Contributions

MK initiated the project and designed the study. MK and MM obtained the hybrid plants. RS and MM performed the identification of molecular markers and made the mitotic chromosome preparations. RS performed the GISH analysis. RS and WU analyzed the microscope images. MK and RS wrote the manuscript. AT supervised the methodology of molecular marker analyses. JN supervised the manuscript.

## Conflict of Interest

The authors declare that the research was conducted in the absence of any commercial or financial relationships that could be construed as a potential conflict of interest.
